# Cervical cancer screening uptake in Arab countries: a systematic review with meta-analysis

**DOI:** 10.1186/s12885-024-13204-7

**Published:** 2024-11-21

**Authors:** Hebatalla Abdelmaksoud Abdelmonsef Ahmed, Mohammed Hamdi Abbas, Hussein Awad Hussein, Rehab Salah Fathy Nasr, Amira Ahmed Lashen, Heba Khaled, Ahmed Azzam

**Affiliations:** 1 Public Health & Community Medicine, Faculty of Medicine, Kafr-Elsheikh University, Kafr-Elsheikh, Egypt; 2 Faculty of Medicine, Tanta University, Tanta, Egypt; 3 Faculty of Medicine, Benha University, Benha, Egypt; 4 Faculty of Pharmacy, Tanta University, Tanta, Egypt; 5 Department of Biochemistry, Faculty of Pharmacy, Cairo University, Cairo, Egypt; 6 Department of Microbiology and Immunology, Faculty of Pharmacy, Helwan University, Cairo, Egypt

**Keywords:** Cervical cancer uptake, Arab countries, Meta-analysis

## Abstract

**Background:**

Cervical cancer, though one of the most common cancers affecting women globally, holds immense potential for prevention through screening. Therefore, we conducted this meta-analysis to assess the rate of cervical cancer screening in Arab countries and identify barriers among those who did not participate.

**Methods:**

A comprehensive search was conducted from January 1st to June 1st,2024, including all observational studies that reported cervical cancer screening uptake in any Arab country. A meta-analysis was performed using a random-effects model to estimate the pooled prevalence, and sensitivity analyses were conducted to test the robustness of the findings. The study followed PRISMA guidelines.

**Results:**

This meta-analysis, covering 55 studies and 204,940 Arab women, found an overall cervical cancer screening uptake rate of 18.2% (95% CI: 13.9–23.6), with sensitivity analysis confirming the reliability of this estimate. Country disparities were evident, with Bahrain having the highest uptake at 44.1%, while Somalia had the lowest at 8.9%. Among women who underwent screening, the majority were ever-married (94.7%) and held positive attitudes towards screening (91.0%). Barriers to screening were common among women who did not participate, with the most frequent reasons being a lack of information (25.1%), the misconception of feeling healthy (24.5%), fear of the procedure (19.3%), and feelings of embarrassment (13.2%). Additionally, women who were screened had lower perceived barrier scores (SMD = -0.466) and higher perceived benefits scores (SMD = 0.379) than those who were not (*p* < 0.05).

**Conclusion:**

This meta-analysis reveals a low overall cervical cancer screening uptake (18.2%) among Arab women. Key barriers such as lack of information, fear, the misconception of feeling healthy, and embarrassment hinder uptake. This alarmingly low rate underscores the urgent need for targeted interventions to address these barriers and promote awareness of early detection’s life-saving potential.

**Supplementary Information:**

The online version contains supplementary material available at 10.1186/s12885-024-13204-7.

## Background

Non-communicable diseases (NCDs) currently account for the majority of global deaths, with cancer expected to be the leading cause of death and the most significant barrier to raising life expectancy in every country in the twenty-first century [1]. One of the most common cancers in women is cervical cancer (CC) [2]. There were around 19 million cases of cancer worldwide in 2020. Of those cases, 600,000 cases were related to CC, accounting for 6.9% of all female cancer cases, despite being one of the most treatable and preventable diseases [3]. Nearly 84% of all cervical malignancies and 88% of all CC fatalities occurred in low-resource countries (those with a Human Development Index (HDI) less than 0.80). The cumulative rates of CC incidence and mortality in high-resource countries were two to four times less than in low-resource countries in 2018 [4].

To eliminate CC, the World Health Organization (WHO) has established goals such as reaching 90% HPV vaccination coverage, guaranteeing that 70% of women get screened at least once by the age of 35 and again by the age of 45, and making sure that 90% of women who are diagnosed with the disease receive treatment [5]. With these goals, we hope to drastically lower the incidence of CC globally [6]. Arab countries exhibit diverse cultures, social structures, and economic backgrounds. Such diversity may manifest in varied healthcare access, awareness, and attitudes toward preventive measures of CC screening [7]. The available data on CC screening prevalence in Arab countries indicate considerable variations across the countries. Factors such as cultural norms, socioeconomic status, education, healthcare infrastructure, and awareness campaigns contribute to the disparities in screening rates [8]. Some Arab countries have made substantial progress in promoting CC screening, with established programs and awareness initiatives contributing to higher prevalence rates [6, 9]. Conversely, other countries face challenges related to limited resources, cultural barriers, and insufficient awareness, resulting in lower screening rates [10].

Ongoing research, systematic reviews, and meta-analyses are essential to providing a comprehensive overview of the current landscape, identifying gaps in knowledge, and informing targeted interventions to improve screening rates and reduce the burden of CC in the Arab world. Therefore, this systematic review and meta-analysis aimed to estimate the pooled CC screening uptake, and commonly reported barriers among women in Arab countries.

## Methods

### Study objectives

The primary aim of this review was to quantify the rate of screening uptake. A secondary aim was to explore the sociodemographic factors of women who underwent CC screening and barriers among women who didn’t. The protocol has been registered with the International Prospective Register of Systematic Review (PROSPERO) (https://www.crd.york.ac.uk/), registration number CRD42023465311.

## Search strategy

A thorough literature search was performed from January 1st to June 1st,2024, using the following databases: PubMed, Scopus, Google Scholar, Web of Science, Cochrane Library, CINAHL Plus, and AMED. Additionally, the reference lists of the eligible articles were reviewed to ensure a thorough representation of the existing literature. This timeframe allows us to analyze data from the recent 20 years, ensuring that our review reflects contemporary practices and trends. Table S1 shows the details of the utilized search strategy. Table S2 shows the 27 items on the PRISMA checklist. The time frame of 2004 to 2024 was selected to allow for the analysis of CC screening practices among women in Arab countries using the most recently available and robust data.

## Eligibility criteria

### Studies were included if they met the following criteria

(1) Observational studies conducted in one of the Arab countries. (2) Studies reported the rate of CC screening based on PAP smear, liquid-based cytology (LBC), Human Papilloma Virus (HPV) testing, or visual inspection on acetic acid (VIA) [11]. (3) All the included studies must be in the English language.

We excluded studies on non-Arab females, non-peer-reviewed articles, and studies with significantly incomplete data.

## Study selection

We initiated the search by entering relevant keywords into the selected databases and creating individual libraries for each database. We removed duplicates using Zotero 6.0. Next, we proceeded with the selection of eligible articles step-by-step. Initially, we excluded articles based on their titles, eliminating those that were deemed completely irrelevant. In cases where there was any doubt, we retained the article for further evaluation in subsequent steps. The remaining articles underwent assessment for eligibility based on their abstract and full text. We also reviewed the reference lists of the eligible articles to identify any potential eligible articles.

### Data extraction

From each study included in the analysis, the following key details were carefully documented by two independent reviewers: last name of the first author, publication time, country, study design, study setting, sample size, age range, CC screening methods, the rate of screening uptake (%), participant characteristics associated with screening uptake (marital status, educational level, knowledge level, attitude level, and perception predictors for CC screening uptake) and reported barriers towards CC screening.

## Quality assessment

We adopted Joanna Briggs Institute’s critical appraisal tool for prevalence studies to assess the quality of the included studies [12]. The Joanna Briggs critical appraisal tool for prevalence studies evaluates nine questions (maximum score is 9) covering three key domains. The first domain relates to the sampling that assesses the appropriateness of the sampling frame, sampling method, sample size, description of the study subjects and setting, and response rate. The second domain is measurement which examines the validity and reliability of the methods used to identify and measure the condition of interest. The third domain focuses on the analysis by assessing the appropriateness of the data analysis methods and the adequacy of the statistical analysis. Two independent reviewers conducted a quality assessment of the included studies, based on the criteria outlined. Any discrepancies between reviewers were resolved through open discussion among all authors. The checklist items are presented in Table S3. Although no specific cutoff values exist to define the overall quality of the study, we categorized studies with scores of 8 and 9 as ‘good,’ those with scores of 6 and 7 as ‘fair,’ and those with scores less than or equal to 5 as ‘low quality.

## Data synthesis

We reported the results as a pooled prevalence with a 95% confidence interval (CI) for CC screening uptake rates and barriers among women who did not undergo the screening. We also presented the pooled standardized mean difference (SMD) in perceived susceptibility and perceived severity between women who participate in the screening and those who don’t.

Subgroup analysis was conducted based on country and study setting (community vs. facility-based study) and specific population categories, such as healthcare workers. The pooled estimates were calculated under the random-effects model. To examine the robustness of the results, sensitivity analysis was performed utilizing the leave-one-out technique. Publication bias testing was not performed as it did not give reliable results in the meta-analysis of proportions [13]. All statistical analyses were performed using Comprehensive Meta-Analysis version 3.0 (Biostat, Englewood, NJ, USA).

**Table 1:** Characteristics of the included studies

Author	Country	Study design	Study setting	Population characteristics	The age range of the studied population	Screening test	Total	Prevalence (%)
**Al-Amro et al.**,** 2020**[14]	Jordan	Cross-sectional	Community-based	General	21–65	Pap test	500	31.2
**Jassim et al.**,** 2018** [15]	Bahrain	Cross-sectional	Facility based	General	-	Pap test	300	40.7
**So et al.**,** 2019**[16]	Kuwait, Oman, Saudi Arabia, and United Arab Emirates	Cross-sectional	Community-based	General	25–64	Pap test	5953	12.7
**Mohamed et al.**,** 2022** [17]	Egypt	Cross-sectional	Facility based	Health care workers	25–70	Pap test	250	44.0
**Al Sharafi & Mohamed**,** 2009** [18]	Kuwait	Cross-sectional	Facility based	General	19–66	Pap test	281	35.2
**Saadoon et al.**,** 2014** [19]	Iraq	Cross-sectional	Facility based	General	30–49	Pap test	222	12.6
**Al Ndour et al.**,** 2012** [2]	Jordan	Cross-sectional	Community-based	General	≥ 35 years	Pap test	1018	27.5
**Al Rifai & Nakamura**,** 2015** [21]	Jordan	Cross-sectional	Community-based	General	20–49	Pap test	8333	25.5
**Bou-Orm et al.**,** 2018** [22]	Lebanon	Cross-sectional	Community-based	General	18–65	Pap test	1813	43.6
**Obeidat et al.**,** 2012** [23]	Jordan	Cross-sectional	Facility based	Healthcare workers	19–68	Pap test	87	19.5
**Al-Meer et al.**,** 2011** [24]	Qatar	Cross-sectional	Facility based	General	≥ 20	Pap test	500	39.4
**Altaian et al.**,** 2019** [25]	Saudi Arabia	Cross-sectional	Community-based	General	18–57	Pap test	450	22.4
**Hesamuddin & Ali S. Daoud.**,** 2016**[26]	Iraq	Cross-sectional	Facility based	General	16–60	Pap test	400	21.5
**Asali & Othman**,** 2022**[27]	Saudi Arabia	Cross-sectional	Community-based	General	21–65	Pap test	434	33.4
**Alwan et al.**,** 2017** [28]	Iraq	Cross-sectional	Facility based	General	-	Pap test	343	8.2
**AMARIN et al.**,** 2008** [29]	Jordan	Cross-sectional	Facility based	General	17–72	Pap test	760	14.3
**Alshamlan et al.**,** 2023** [30]	Saudi Arabia	Cross-sectional	Facility based	Healthcare workers	24–65	Pap test & HPV DNA testing	1008	24.6
**Hasan et al.**,** 2021** [31]	Iraq	Cross-sectional	Community-based	General	19–55	Pap test	530	25.3
**Alzahrani et al.**,** 2018** [32]	Saudi Arabia	Cross-sectional	Facility based	General	20–60	Pap test	380	29.7
**Fadhil et al.**,** 2008** [33]	Bahrain	Cross-sectional	Community-based	General	-	Pap test	350	47.4
**Elgamal**,** 2015** [34]	Egypt	Cross-sectional	Facility based	Healthcare workers	20–60	Pap test	150	6.7
**Zahid et al.**,** 2022** [35]	Saudi Arabia	Cross-sectional	Community-based	General	≥ 18	Pap test	1498	12.5
**Abdulmalek & Kalari**,** 2019** [36]	Iraq	Cross-sectional	Facility based	General	21–65	Pap test	400	9.5
**Altunkurek al.**,** 2022**[37]	Somalia	Cross-sectional	Facility based	Health care workers	-	Pap test	147	2.0
**Daher**,** 2019** [38]	Saudi Arabia	Cross-sectional	Community-based	General	15–65	Pap test	255	0.8
**Alshahrani MS**,** 2020**[39]	Saudi Arabia	Cross-sectional	Community-based	General	18–50	Pap test	1990	14.2
**Mwanje J**,** 2023**[40]	Sudan	Cross-sectional	Community-based	General	26–65	Pap test	575	11.5
**Barghouti FF**,** 2008** [41]	Jordan	Cross-sectional	Facility based	General	> 17	Pap test	674	40.3
**Ibrahim et al.**,** 2022** [42]	Saudi Arabia	Cross-sectional	Community-based	General	18–65	Pap test	1085	2.0
**Al-Attar et al.**,** 2014** [43]	Iraq	Cross-sectional	Facility based	Healthcare workers	-	Pap test	56	7.2
**Heena et al.**,** 2019** [44]	Saudi Arabia	Cross-sectional	Facility based	Healthcare workers	-	Pap test	395	26.2
**Yahya et al.**,** 2021** [45]	Oman	Cross-sectional	Community based	General	≥ 18	Pap test	805	15.7
**Bencher et al.**,** 2022** [46]	Algeria	Cross-sectional	Community-based	General	-	Pap test	715	1.2
**Al Kalbani et al.**,** 2022** [47]	Oman	Cross-sectional	Facility based	General	19–75	Pap test	285	40.0
**El-Hammadi et al.**,** 2009** [48]	Kuwait	Cross-sectional	Community-based	General	-	Pap test	299	44.0
**Al Eyd & Shaik**,** 2012** [49]	United Arab Emirates	Retrospective study	Facility based	General	20–71	Pap test	150,111	0.4
**Al-Mubarak et al.**,** 2016** [50]	Sudan	Cross-sectional	Community-based	General	14–58	Pap test	500	15.8
**Annaisha et al.**,** 2019** [51]	Saudi Arabia	Cross-sectional	Community-based	General	15–65	Pap test	2220	15.3
**Rezq et al.**,** 2023**[52]	Saudi Arabia	Cross-sectional	Community-based	General	18–50	Pap test	421	8.3
**Walz et al.**,** 2022** [53]	Somalia	Cross-sectional	Facility based	Healthcare workers	-	Pap test	404	11.0
**Walz et al.**,** 2022**[53]	Somalia	Cross-sectional	Facility based	Healthcare students	-	Pap test	404	15.0
**Arechkik et al.**,** 2023** [54]	Morocco	Cross-sectional	Facility based	HIV-positive women	20–78	Pap test & VIA	494	44.7
**Ghamdi**,** 2022** [55]	Saudi Arabia	Cross-sectional	Community-based	General	≥ 18	Pap test	755	21.1
**Akkour et al.**,** 2021**[56]	Saudi Arabia	Cross-sectional	Community-based	General	-	-	564	8.0
**Bendahhou et al.**,** 2023** [57]	Morocco	Cross-sectional	Facility based	General	≥ 18	Pap test & VIA	400	28.0
**Gamaoun**,** 2018** [58]	Tunisia	Cross-sectional	Facility based	General	-	Pap test	452	27.7
**Alshammiri**,** 2022** [59]	Saudi Arabia	Cross-sectional	Facility based	General	-	Pap test	386	5.7
**Telvizian et al.**,** 2021** [60]	Lebanon	Cross-sectional	Facility based	General	-	Pap test	262	36.7
**Telvizian et al.**,** 2021** [60]	Lebanon	Cross-sectional	Community based	General	-	Pap test	407	44.5
**Alshehri H et al.**,** 2024** [61]	Saudi Arabia	Cross-sectional	Community based	General	21–65	Pap test	127	30.7
**Alshehri H et al.**,** 2024** [61]	Saudi Arabia	Cross-sectional	Facility based	Healthcare workers	21–65	Pap test	126	20.6
**Alfareh M et al.**,** 2024** [62]	Saudi Arabia	Cross-sectional	Community based	General	-	Pap test	8194	22.5
**M Al Kindi R et al.**,** 2024** [63]	Oman	Cross-sectional	Facility based	General	18–50	Pap test	380	21
**Atef S et al.**,** 2024** [64]	Egypt	Cross-sectional	Facility based	General	18–50	Pap test	376	70.5
**Alkhamis et al.**,** 2023** [65]	Saudi Arabia	Cross-sectional	Community based	General	21–65	Pap test	2,337	22.1%
**Elbarazi et al.**,** 2023** [66]	United Arab Emirates	Cross-sectional	Facility-based	General	> 30	Pap test	300	31.3%
**Prashanth et al.**,** 2023**[67]	United Arab Emirates	Cross-sectional	Facility-based	General	> 20	Pap test	401	31.2%
**Al-Shamsi et al.**,** 2023**[68]	United Arab Emirates	Cross-sectional	Community based	General	21–60	Pap test	1,378	12.8%
***HPV: human papillomavirus***,*** VIA: Visual Inspection on Acetic Acid***								

**Table 2:** Meta-analysis of the prevalence of cervical cancer screening in arab countries:

Study group	Included studies& citations	Total sample size	Pooled prevalence (%), 95% CI	I squared (%)	Q-value, *P*-value
Overall	55	204,940	18.2 (13.9:23.6)	99.6%	13,876, *P* < 0.001
**Based on country**					
Saudi Arabia	17 [25] [27] [30] [32] [35] [38] [39] [42] [44] [51] [6] [55] [56] [59] [61] [62][65]	22,625	15.8(13.1: 19)	96.8%	542, *P* < 0.001
Jordan	6 [14] [20] [21] [23] [29] [41]	11,372	26.3(20.5:32.2)	96.4%	131.1, *P* < 0.001
Iraq	6 [19] [26] [28] [31] [36] [43]	1,951	13.4(8.5:20.4)	93.5%	70.6, *P* < 0.001
United Arab Emirates	4 [49][66][67][68]	152,723	13.1(1.3: 62.8)	99.9	5174, *P* < 0.001
Egypt	3 [17] [34] [64]	776	34.4(11.9:67.1)	98.3	117.1, *P* < 0.001
Kuwait	2 [18] [48]	880	39.7(31.3:48.7)	79.1%	4.7, *P* = 0.029
Oman	3 [45] [47] [63]	1,470	24.2(13:40.6)	97.1%	67.6, *P* < 0.001
Bahrain	2 [15] [33]	650	44.1(37.6:50.8)	66.5	2.98, *P* = 0.08
Somalia	2 [37] [53]	955	8.9(3.3:16.7)	92.4%	26.2, *P* < 0.001
Lebanon	2 [22] [60]	2,482	42.2(38.5:46.1)	59.2%	4.9, *P* = 0.08
Sudan	2 [40] [50]	1,075	13.5 (9.8:18.3)	76.5%	4.2, *P* = 0.04
Morocco	2 [54] [57]	894	18.9(1.7:76.1)	99.1%	131.8, *P* < 0.001
Healthcare workers	9 [17] [23] [30] [34] [37] [43] [44] [53] [61]	3027	16(11: 22.6)	94.1	153.9, *P* < 0.001
**Based on the study setting**					
Community-based	28 [14] [21] [22] [25] [27] [31] [33] [35] [38] [39] [40] [42] [45] [46] [48] [50] [51] [52] [55] [56] [60] [61] [62]	44,300	19.2(16.1:22.7)	98.5%	1962.2, *P* < 0.001
Facility-based	30 [15] [17] [18] [19] [23] [24] [26] [28] [29] [30] [32] [34] [36] [37] [41] [43] [44] [47] [49] [53] [54] [57] [58] [59] [60] [61] [63] [64]	160,640	18.1(9.1:32.8)	99.7%	6819.1, *P* < 0.001

**Table 3:** Meta-analysis of sociodemographic factors of women who underwent cervical cancer screening in arab countries

Study group	Included studies& citations	Total sample size	Pooled prevalence (%), 95% CI	I-squared (%)	Q-value, *P*-value
Based on marital status					
Ever Married	8 [16] [22] [27] [31] [32] [36] [45] [47]	2218	94.7(79.0:98.8)	97.3%	264.8, *P* < 0.001
Single			5.3(1.2:21.0)		
**Based on educational status**					
Higher level	13 [16] [19] [20] [21] [22] [27] [30] [31] [32] [36] [45] [47] [55]	5058	52.3(29.1:75.4)	99.3%	1,740.3, *P* < 0.001
Lower level			47.7(24.6:70.9)		
**Based on the knowledge level**					
Good/moderate knowledge	5 [19] [26] [27] [36] [55]	456	64.7(35.3:86.1)	96.1%	104.5, *P* < 0.001
Poor knowledge			35.3(13.9:64.7)		
**Based on attitude level**					
Positive attitude	3 [19] [36] [55]	225	91.0(86.0:97.9)	86.2%	14.5, *P* = 0.001
Neutral or Negative attitude			9.0(2.1:32.0)		

**Table 4:** Meta-analysis of the barriers to cervical cancer screening uptake among women who did not undergo cervical cancer uptake

Studied Barriers	Included studies& citations	Pooled prevalence (%), 95%CI	I squared (%)	Q-value, *P*-value
**Consider as healthy**	10 [23] [26] [27] [28] [29] [31] [37] [38] [44] [57]	24.5(19.0:31.1)	91.8%	109.9, *P* < 0.001
**Fear of screening**	16 [25] [26] [27] [28] [29] [30] [35] [36] [38] [43] [44] [45] [46] [47] [51] [57]	19.3(12.7:28.4)	98.3%	900, *P* < 0.001
**Lack of information**	14 [26] [29] [30] [31] [35] [36] [37] [43] [44] [46] [47] [51] [57] [62]	25.1(14.8:39.3)	99.5%	2288.8, *P* < 0.001
**Embarrassment**	17 [14] [23] [25] [26] [27] [28] [29] [30] [31] [36] [37] [38] [46] [47] [51] [57] [62]	13.2(7.8 :21.5)	98.1%	1598.1, *P* < 0.001

## Results

### Study identification and characteristics of included studies

**Fig. 1:**
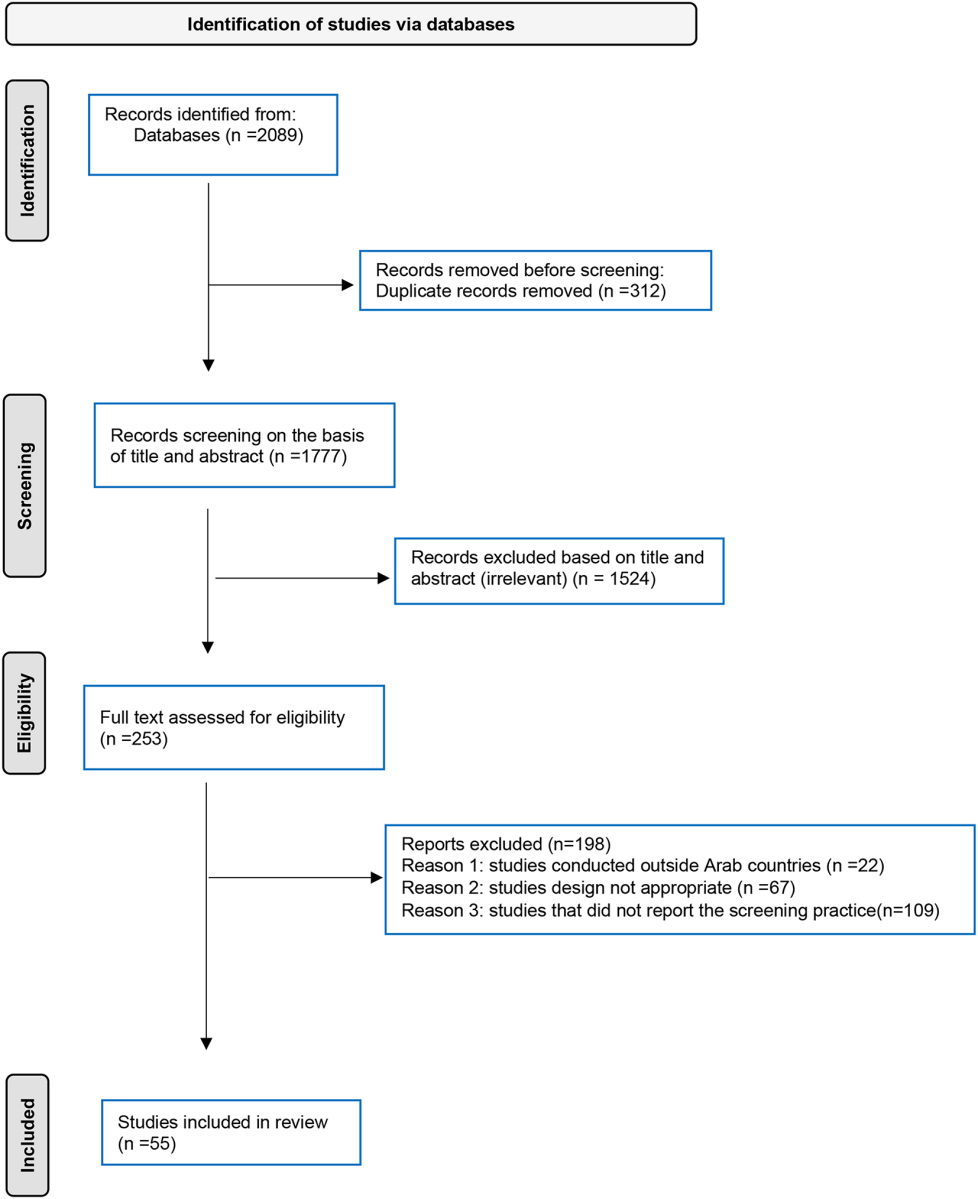
Flow chart depicting the selection of publications

This systematic review and meta-analysis encompassed all published studies on CC screening uptake in Arab countries, incorporating a total of 2089 articles. Of these, 312 duplicate records were removed, and 1777 articles were excluded by screening using their titles and abstracts. Subsequently, 253 full-text papers were assessed for eligibility based on the inclusion and exclusion criteria. Finally, 55 studies were included in the final quantitative meta-analysis, as depicted in Fig. 1. The population of interest in these studies ranged from general women to specific subgroups, such as female university students, female healthcare workers, and women with particular health concerns.

### Study selection and characteristics of included studies

Table 1 shows the characteristics of the eligible articles. Overall, 55 studies with 204,940 Arabian women were included. From this, 30 studies were facility-based cross-sectional studies (FBCS), and 28 were community-based cross-sectional studies (CBCS). The largest sample size was 150,111 women in the United Arab Emirates [49]. The smallest sample was 56 women from a study conducted among healthcare workers in Iraq [43].

All studies were conducted in fifteen Arabic countries. Seventeen studies were from Saudi Arabia, six studies were from Jordan and Iraq, four studies from the United Arab Emirates, three studies were from Oman and Egypt, two studies were from Kuwait, Somalia, Morocco, Sudan, Bahrain, and Lebanon, and only one study was from Qatar, Tunisia, and Algeria. One study was also conducted in four Gulf countries (Kuwait, Oman, Saudi Arabia, and the United Arab Emirates). Nine studies were conducted among healthcare workers or students, and only one study was among HIV-positive women.

### The overall cervical cancer uptakes among arabs

**Fig. 2:**
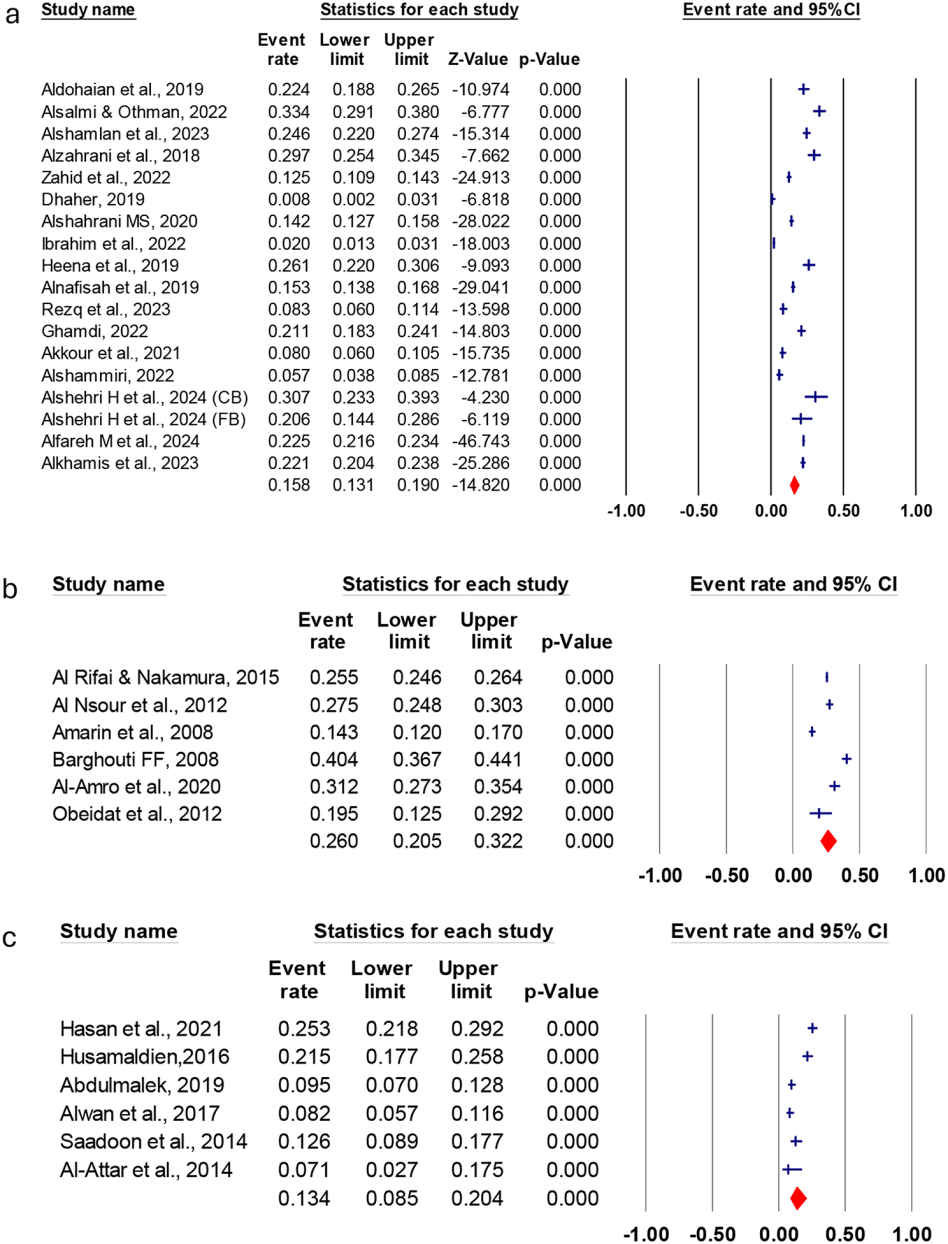
Association of perceived susceptibility to cervical cancer. Meta-Analysis of Cervical Cancer Screening Uptake Among Women in (**a**) Saudi Arabia, (**b**) Jordan, and (**c**) Iraq

Table 2 shows the pooled rate of CC screening uptake, with heterogeneity statistics. The meta-analysis of 55 studies showed that the pooled Arabian CC screening uptake was 18.2% (95% CI: 13.9%:23.6%), as depicted in Fig. S1. The heterogeneity was high, as evidenced by the I^2^% value of 99.6% presented in Table 2. We conducted the subgroup analysis based on the country, study setting, and specific population category, such as healthcare workers. The meta-analysis based on the country revealed that the highest CC screening uptake was observed in Bahrain at 44.1% (95% CI: 37.6%:50.8%) followed by Lebanon at 42.2% (95% CI: 38.5%:46.1%) and the lowest occurred in Somalia 8.9% (95% CI: 3.3%:16.7%). The CC screening rates were 15.8% (95% CI: 13.1%: 19.0%) in Saudi Arabia, 26.3% (95% CI: 20.5%: 32.2%) in Jordan, and 13.4% (95% CI: 8.5%:20.4%) in Iraq, as illustrated in Fig. 2.

Regarding the study setting, the pooled subgroup analysis showed that CC screening was 19.2(95% CI: 16.1:22.7) in community-based cross-sectional studies, compared to 18.1(95% CI: 9.1:32.8) for facility-based studies. Among healthcare workers or students, CC uptake was 16.0% (95% CI: 11.0%: 22.6%).

### Sociodemographic factors of women who underwent cervical cancer screening

Table 3 presents the sociodemographic factors associated with CC screening uptake. The uptake rate for ever-married women was 94.7% (95% CI: 79.0%: 98.8%), while single women had a much lower uptake rate of 5.3% (95% CI: 1.2–21.0%). while women with higher education levels had a comparable uptake rate (52.3%, 95% CI: 29.1%: 75.4%) to those with lower education levels (47.7, 95% CI: 24.6%: 70.9%). Those with good to moderate knowledge have an uptake rate of 64.7% (95% CI: 35.3%:86.1%) compared to 35.3% (95% CI: 13.9%:64.7%) with low knowledge. For women with a positive attitude, the uptake rate was 91.0% (95% CI: 86.0%:97.9%) compared to 9.0% (95% CI: 2.1%:32.0%) for women with a negative attitude.

### Perceived susceptibility and severity for women who undergo cervical cancer screening compared to those who do not

**Fig. 3:**
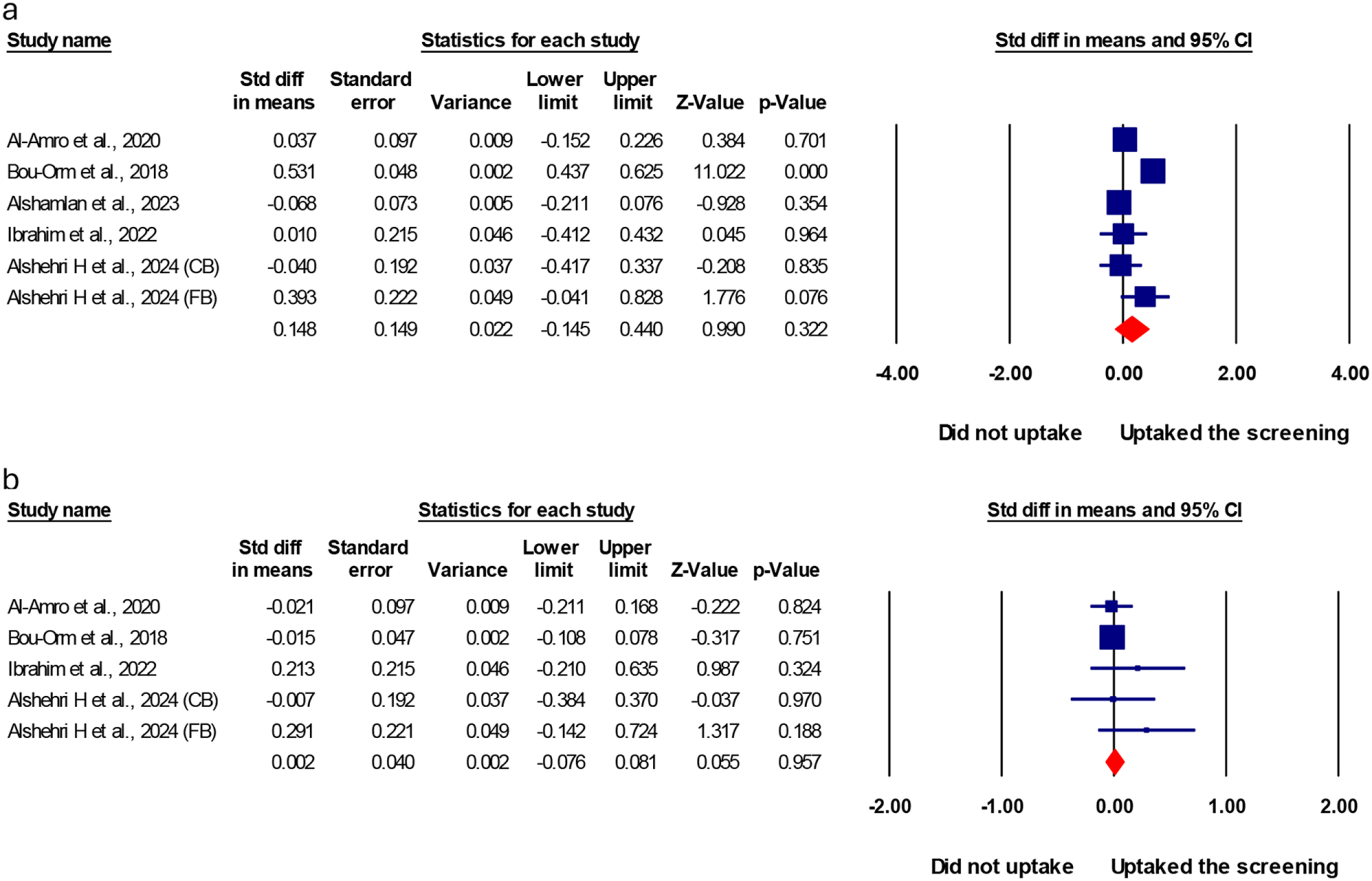
Association of perceived severity to cervical cancer. Meta-analysis of the standardized mean difference (SMD) of perceived susceptibility and severity for women who undergo cervical cancer screening compared to those who do not. **a**; pooled SMD of perceived susceptibility; **b**; pooled SMD of perceived severity

There wasn’t a statistically significant difference in standardized mean scores of perceived susceptibility and perceived severity towards CC in women who uptake CC screening compared to those who did not (SMD = 0.148, (95% CI: (-)0.145: 0.440) and (SMD = 0.002, (95% CI: (-)0.076: 0.081), respectively, as shown in Fig. 3.

### Perceived barriers and benefits for women who undergo cervical cancer screening compared to those who do not

**Fig. 4:**
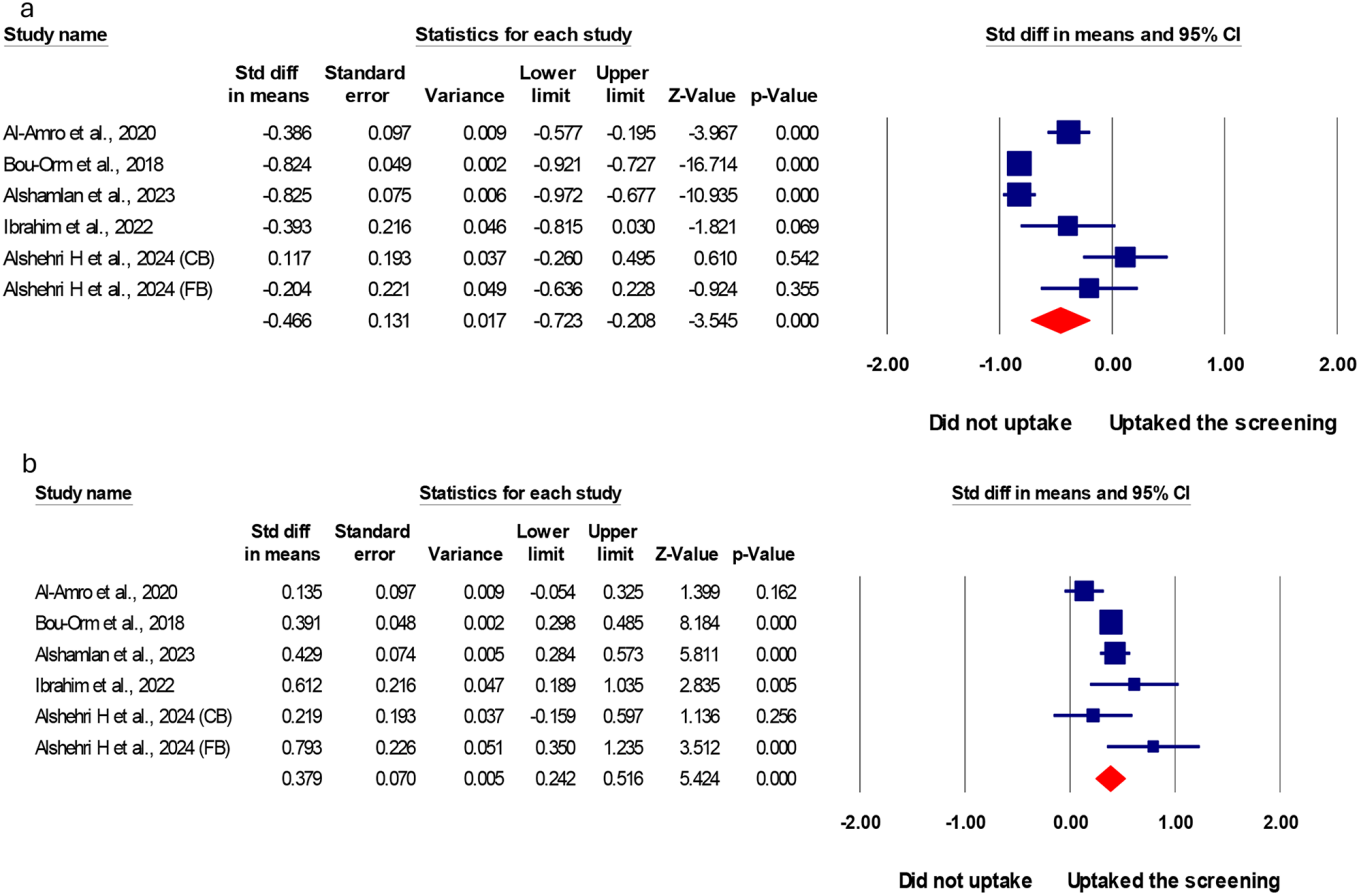
Meta-analysis of the standardized mean difference (SMD) of perceived barriers and benefits for women who undergo cervical cancer screening compared to those who do not. **a**; pooled SMD of perceived barriers, **b**; pooled SMD of perceived benefits

The pooled effect of five studies ( [14]^,^ [22]^,^ [30]^,^ [42]^,^ [61]) revealed that the perceived barriers to CC screening were major predictors of CC screening uptake in Arab countries. Women who did not undergo CC screening had higher perceived barriers scores towards CC screening than their counterparts (SMD = (-)0.466, (95% CI: (-)0.732: (-)0.208)) (Fig. 3). On the contrary, women who underwent CC screening had higher perceived benefits scores for CC screening than their counterparts (SMD = 0.379, 95% CI: 0.242:0.516) (Fig. 4).

### Barriers to cervical cancer screening uptake

Among those who did not undergo the screening, the most common barriers that hindered the uptake of CC screening were lack of information 25.1% (95% CI:14.8%:39.3%), followed by considered as healthy 24.5% (95% CI: 19.0%:31.1%), fear of screening 19.3% (95% CI: 12.7%:28.4%) and embarrassment 13.2%(95% CI: 7.8%:21.5%) (Table 4).

### Sensitivity analysis

We conducted a sensitivity analysis using the leave-one-out method, which demonstrated that the overall estimate did not deviate by more than 0.7% when any individual study was excluded, except for a single study from the United Arab Emirates. This study accounted for 73.2% of the total sample. Excluding this study resulted in a more precise 95% confidence interval, with a pooled CC screening uptake rate of 20.2% (95% CI: 17.7 to 23.0), compared to the overall analysis, which indicated a pooled uptake rate of 18.2% (95% CI: 13.9 to 23.6), as shown in Fig. S2.

## Discussion

CC, though one of the most common cancers affecting women globally, holds immense potential for prevention [69]. With well-structured screening programs and widespread vaccination, a staggering 95% of its burden could be mitigated. Embracing this vision, the World Health Organization (WHO) launched a global strategy to combat CC, aiming to reduce its incidence significantly. A key objective of this strategy is to ensure that 70% of women undergo screening for pre-cancerous lesions by 35 and again by 45 [70]. To assess the progress towards this vital target, we conducted a systematic review and meta-analysis, unveiling current CC screening rates in Arab countries. Overall, our findings reveal a low level of utilization of CC screening among Arabians, at 18.2%. The most common factors associated with a higher CC screening rate are married women, positive attitudes, perceptions of fewer barriers, and more benefits. These findings highlight the overall low uptake of CC screening in the Arab population.

When compared to other studies, the utilization rate of CC screening among individuals from Arabian countries (17.6%) is much lower than in Europe and the USA, estimated to range between 50% and 80% [71–75]. In contrast, it is relatively higher than the uptake of CC screening in Sub-Saharan Africa, which was reported at 12.8% (95% CI: 10.2: 15.5) [76]. Our findings revealed significant differences in CC screening utilization by country. Bahrain had a screening rate of 44.1%, whereas Somalia had a much lower rate of 8.9% (95% CI: 3.3%:16.7%), respectively. These findings may reflect differences in awareness campaigns, cultural factors, or other contextual factors that impact screening practices in each country.

In this study, it was found that most women who underwent the screening were ever-married, at 94.7%. This finding aligns with recent studies conducted in Ethiopia and Ghana, which demonstrated that married women were more likely to undergo CC screening compared to unmarried women, with adjusted odds ratios of 10.74 and 3.98, respectively [77, 78]. The increased uptake of screening among married women may be attributed to various factors. One possible reason is spousal support and encouragement, as married women may receive backing and motivation from their husbands to prioritize preventive healthcare. Additionally, cultural and religious misconceptions surrounding premarital relationships, virginity, and gynecological examinations might act as barriers for unmarried women, deterring them from seeking screening or limiting their independent access to healthcare. The stigma associated with screening may be another factor that was reported not only in low-income countries but also in high-income countries. For instance, a study conducted in London highlighted that Muslim woman expressed hesitancy towards screening due to feelings of embarrassment and fear. These concerns stemmed from their unmarried status and the potential perception that the screening implies sexual activity [79]. A further study carried out in the United Kingdom uncovered that the acceptance of CC screening was marred by stigma, primarily because of its association with human papillomavirus (HPV) and the belief that it implies women’s negligence towards their health responsibilities [80].

Most women who underwent the screening had a positive attitude at 91.0% (95% CI: 86.0%:97.9%). This suggests that attitudes and perceptions towards screening play a crucial role in motivating women to participate in the screening program. However, there was an inconsistency observed when considering the educational level and knowledge level of the participants. The overlap of 95% confidence intervals suggests that there is no statistically significant difference in screening uptake based on educational level or knowledge level. This may be related to the small number of included studies (5 studies).

Additionally, this study demonstrates that women who didn’t uptake CC screening perceived barriers to CC screening more than their counterparts (SMD= (-)0.466, (95% CI: (-)0.732: (-)0.208, *P* < 0.05). On the other side, women who underwent CC screening had a higher perceived benefit score towards CC screening than those who did not undergo the screening (SMD = 0.379, 95% CI: 0.242:0.516, *P* < 0.05). The most prevalent barriers among women who didn’t undergo previous CC screening were lack of information, considering themselves to be healthy, fear of screening, and embarrassment, with overall prevalence at 25.1%, 24.5%, 19.3%, and 13.2%, respectively. Similarly, a previous systematic review examining barriers to CC screening in Arab countries identified limited knowledge and awareness among Arab women, concerns about pain and embarrassment, stigma, and sociocultural beliefs as key factors affecting screening uptake [81]. Another significant obstacle to CC screening in Arab countries is the lack of structured, population-based programs, leading to screening often being conducted opportunistically [81]. This reliance on incidental, provider-initiated screening is further hindered by limited provider knowledge in these regions [17, 37, 82].

The implications of these findings underscore the need for policymakers to prioritize the establishment of organized and accessible CC screening programs, as effective interventions to improve screening rates must address both the barriers to, and the perceived benefits of, participation. A dual-faceted approach is recommended: initially, efforts should aim to mitigate perceived barriers by increasing public awareness about the accessibility and affordability of screening services, addressing concerns related to stigma, embarrassment, and cancer-related anxieties, and improving convenience through flexible scheduling and enhanced clinic accessibility. Equally important is the need to amplify perceived benefits. Educational campaigns should underscore the critical role of screening in CC prevention, the value of early detection and treatment, and the reassurance that regular checkups provide. Additional interventions, such as electronic reminders, community-based self-sampling for HPV testing, and free or subsidized screening services, have also demonstrated efficacy in promoting higher screening uptake [83].

### Study limitations

It is important to acknowledge the limitations inherent in our research. One notable limitation is the absence of studies in several countries, including Comoros, Djibouti, Libya, Mauritania, Palestine, Syria, and Yemen, which may restrict the generalizability of the study findings. In addition, statistical heterogeneity is inevitable in a meta-analysis of epidemiology. While most publications in the medical field are in English, and many high-quality databases primarily require publications in English, a potential bias may be introduced by including only studies published in English. Finally, there is an absence of intervention studies that aim to improve the uptake of CC screening. These identified limitations emphasize the importance of future research endeavors that address these specific points.

## Conclusion

CC is one of the most prevalent cancers affecting women worldwide, despite being largely preventable through early detection and screening. This meta-analysis, encompassing 55 studies and 204,940 Arab women, reveals an alarmingly low screening uptake rate of only 18.2%. Key barriers such as lack of information, fear, the misconception of feeling healthy, and embarrassment hinder uptake. These findings highlight the urgent need for targeted interventions designed to increase awareness of the life-saving benefits of early detection and address the specific cultural and information barriers preventing wider adoption of screening practices.

## Electronic supplementary material

Below is the link to the electronic supplementary material.

Supplementary Material 1

## Data Availability

All data generated or analyzed during this study were included in this published article in its supplementary information file.

## Abbreviations

NCDs: Non-communicable diseases
HDI: Human Development Index
WHO: World Health Organization
HPV: Human Papilloma Virus
LBC: Liquid-Based Cytology
VIA: Visual Inspection on Acetic Acid
