# Correction: Cervical cancer screening uptake in Arab countries: a systematic review with meta-analysis

**DOI:** 10.1186/s12885-024-13233-2

**Published:** 2024-11-29

**Authors:** Hebatalla Abdelmaksoud Abdelmonsef Ahmed, Mohammed Hamdi Abbas, Hussein Awad Hussein, Rehab Salah Fathy Nasr, Amira Ahmed Lashen, Heba Khaled, Ahmed Azzam

**Affiliations:** 1 Public Health & Community Medicine, Faculty of Medicine, Kafr-Elsheikh University, Kafr-Elsheikh, Egypt; 2 Faculty of Medicine, Tanta University, Tanta, Egypt; 3 Faculty of Medicine, Benha University, Benha, Egypt; 4 Faculty of Pharmacy, Tanta University, Tanta, Egypt; 5 Department of Biochemistry, Faculty of Pharmacy, Cairo University, Cairo, Egypt; 6 Department of Microbiology and Immunology, Faculty of Pharmacy, Helwan University, Cairo, Egypt

**Correction: BMC Cancer 24, 1438 (2024)**

10.1186/s12885-024-13204-7

Following publication of the original article [1], the authors have reported an error in the given name of author Hussein Awad Hussein.

The incorrect given name is: Hussein Awed.

The correct given name is: Hussein Awad.

Further to this, Hussein Awad Hussein is affiliated to institution 2, Faculty of Medicine, Tanta University, Tanta, Egypt.

The details in this correction article are correct and the original article [1] has been updated.
