# Supplementary material for: Cervical cancer screening uptake in Arab countries: a systematic review with meta-analysis
Source: BMC Cancer. 2024 Nov 21;24:1438. doi: 10.1186/s12885-024-13204-7 (PMC11583763; doi:10.1186/s12885-024-13204-7)
Supplement: Supplementary file 1 — Supplementary Material 1 [file 12885_2024_13204_MOESM1_ESM.docx]

**Supplementary file**

**Cervical Cancer Screening Uptake in Arab Countries: A Systematic Review with Meta-Analysis**

**Hebatalla Abdelmaksoud Abdelmonsef Ahmed^1*^, Mohammed Hamdi Abbas^2^, Hussein Awed Hussein^3^, Rehab Salah Fathy Nasr^4^,** **Amira Ahmed lashen^5^,** **Heba Khaled^6^,** **Ahmed Azzam ^7^**

^1^ Public Health & Community Medicine, Faculty of Medicine, Kafr-Elsheikh University, Egypt.

Email addresses: [Heba_Abdelmasoud@med.kfs.edu.eg](mailto:Heba_Abdelmasoud@med.kfs.edu.eg).

<https://orcid.org/0000-0002-1882-6623>

^2^ Faculty of Medicine, Tanta University, Egypt.
Email addresses: [mhamdi2204@gmail.com](mailto:mhamdi2204@gmail.com)

^3^ Faculty of Medicine, Tanta University, Egypt.

Email addresses: husseinawad505@gmail.com

^4^ Faculty of Medicine, Benha University, Egypt.

Email addresses: rehabnasr26@gmail.com

^5^ Faculty of Pharmacy, Tanta University, Egypt.

Email addresses: e.ahmed9080@gmail.com

^6^ Department of Biochemistry, Faculty of Pharmacy, Cairo University, Cairo, Egypt.

Email addresses: heba.kaled.fathalla@std.pharma.cu.edu.eg

^7^ Department of Microbiology and Immunology, Faculty of Pharmacy, Helwan University, Cairo Egypt.

Email: ahmed.abdelkareem@pharm.helwan.edu.eg

*The corresponding author

Hebatalla Abdelmaksoud Abdelmonsef Ahmed
Public Health & Community Medicine, Faculty of Medicine, Kafr-Elsheikh University, Egypt.

Email addresses: Heba_Abdelmasoud@med.kfs.edu.eg.

https://orcid.org/0000-0002-1882-6623

| **Search** | **Search terms** |
| --- | --- |
| **#1** | **(Cervical cancer) OR (cervical neoplasm) OR (cervical tumor)** |
| **#2** | **(screening) OR (preclinical)** |
| **#3** | **(uptake) OR (utilize) OR (practice)** |
| **#4** | **((Algeria) OR (Bahrain) OR (Comoros) OR (Djibouti) OR (Egypt) OR (Iraq) OR (Jordan) OR (Kuwait) OR (Lebanon) OR (Libya) OR (Mauritania) OR (Morocco) OR (Oman) OR (Palestine) OR (Qatar) OR (Saudi Arabia) OR (Somalia) OR (Sudan) OR (Syria) OR (Tunisia) OR (United Arab Emirates) OR (Yemen) OR (Arab))** |
| **#5** | **#1 AND #2** **AND #3 AND #4** |
| **#6** | **Limit #6 in English.** |

**Table S1: Comprehensive elements of the PubMed search strategy**

**Table S2: Supplementary preferred reporting items for systematic reviews and meta-analyses (PRISMA) checklist.**

| Section/topic | Item No | Checklist item | Reported on page No |
| --- | --- | --- | --- |
| Title | 1 | Identify the report as a systematic review, meta-analysis, or both | 1 |
| Abstract | | | |
| Structured summary | 2 | Provide a structured summary including, if applicable, background, objectives, data sources, study eligibility criteria, participants, interventions, study appraisal and synthesis methods, results, limitations, conclusions and implications of key findings, systematic review registration number | 2,3 |
| Introduction | | | |
| Rationale | 3 | Describe the rationale for the review in the context of what is already known | 4 |
| Objectives | 4 | Provide an explicit statement of questions being addressed with reference to participants, interventions, comparisons, outcomes, and study design (PICOS) | 4 |
| Methods | | | |
| Protocol and registration | 5 | Indicate if a review protocol exists, if and where it can be accessed (such as web address), and, if available, provide registration information including registration number | 4 |
| Eligibility criteria | 6 | Specify study characteristics (such as PICOS, length of follow-up) and report characteristics (such as years considered, language, publication status) used as criteria for eligibility, giving rationale | 5 |
| Information sources | 7 | Describe all information sources (such as databases with dates of coverage, contact with study authors to identify additional studies) in the search and date last searched | 5 |
| Search | 8 | Present a full electronic search strategy for at least one database, including any limits used, such that it could be repeated | 5  Table S1 |
| Study selection | 9 | State the process for selecting studies (that is, screening, eligibility, included in the systematic review, and, if applicable, included in the meta-analysis) | 5 |
| Data collection process | 10 | Describe the method of data extraction from reports (such as piloted forms, independently, in duplicate) and any processes for obtaining and confirming data from investigators | 5 |
| Data items | 11 | List and define all variables for which data were sought (such as PICOS, and funding sources) and any assumptions and simplifications made | 5 |
| Risk of bias in individual studies | 12 | Describe methods used for assessing the risk of bias in individual studies (including specification of whether this was done at the study or outcome level), and how this information is to be used in any data synthesis | 6  Table S3, S4 |
| Summary measures | 13 | State the principal summary measures (such as risk ratio, and difference in means). | 6 |
| Synthesis of results | 14 | Describe the methods of handling data and combining results of studies, if done, including measures of consistency (such as I^2^ statistic) for each meta-analysis | 6 |
| Risk of bias across studies | 15 | Specify any assessment of risk of bias that may affect the cumulative evidence (such as publication bias, selective reporting within studies) | 7 |
| Additional analyses | 16 | Describe methods of additional analyses (such as sensitivity or subgroup analyses, meta-regression), if done, indicating which were pre-specified | 7 |
| Results | | | |
| Study selection | 17 | Give numbers of studies screened, assessed for eligibility, and included in the review, with reasons for exclusions at each stage, ideally with a flow diagram | Fig.1 |
| Study characteristics | 18 | For each study, present characteristics for which data were extracted (such as study size, PICOS, follow-up period) and provide the citations | 7,8  Table 1 |
| Risk of bias within studies | 19 | Present data on the risk of bias of each study and, if available, any outcome-level assessment (see item 12). | - |
| Results of individual studies | 20 | For all outcomes considered (benefits or harms), present for each study (a) simple summary data for each intervention group and (b) effect estimates and confidence intervals, ideally with a forest plot | 12-14  Figs. 2-5 and Tables 2-4 |
| Synthesis of results | 21 | Present results of each meta-analysis done, including confidence intervals and measures of consistency | 12-14  Figs. 2-5 and Tables 2-4 |
| Risk of bias across studies | 22 | Present results of any assessment of the risk of bias across studies (see item 15) | - |
| Additional analysis | 23 | Give results of additional analyses, if done (such as sensitivity or subgroup analyses, meta-regression) (see item 16) | 14, Fig. S1 |
| Discussion | | | |
| Summary of evidence | 24 | Summarize the main findings including the strength of evidence for each main outcome; consider their relevance to key groups (such as health care providers, users, and policymakers) | 14-17 |
| Limitations | 25 | Discuss limitations at the study and outcome level (such as the risk of bias), and at the review level (such as incomplete retrieval of identified research, reporting bias) | 17,18 |
| Conclusions | 26 | Provide a general interpretation of the results in the context of other evidence, and implications for future research | 18 |
| Funding | | | |
| Funding | 27 | Describe sources of funding for the systematic review and other support (such as the supply of data) and the role of funders for the systematic review | 19 |

**Table S3: The checklist items for Joanna Briggs's critical appraisal tool for prevalence studies.**

|  | Yes | No | Unclear | Not applicable |
| --- | --- | --- | --- | --- |
| 1. Was the sample frame appropriate to address the target population? | □ | □ | □ | □ |
| 1. Were study participants sampled in an appropriate way? | □ | □ | □ | □ |
| 1. Was the sample size adequate? | □ | □ | □ | □ |
| 1. Were the study subjects and the setting described in detail? | □ | □ | □ | □ |
| 1. Was the data analysis conducted with sufficient coverage of the identified sample? | □ | □ | □ | □ |
| 1. Were valid methods used for the identification of the condition? | □ | □ | □ | □ |
| 1. Was the condition measured in a standard, reliable way for all participants? | □ | □ | □ | □ |
| 1. Was there appropriate statistical analysis? | □ | □ | □ | □ |
| 1. Was the response rate adequate, and if not, was the low response rate managed appropriately? | □ | □ | □ | □ |

**Table S4: Quality assessment of the included studies.**

| **Author** | **Checklist item** | | | | | | | | | **Score**  **(Out of 9)** |
| --- | --- | --- | --- | --- | --- | --- | --- | --- | --- | --- |
|  | **1** | **2** | **3** | **4** | **5** | **6** | **7** | **8** | **9** |  |
| **Al-Amro et al., 2020**[1] | 1 | 0 | 0 | 1 | 1 | 1 | 1 | 1 | 1 | **7** |
| **Jassim et al., 2018** [2] | 1 | 1 | 1 | 1 | 1 | 1 | 1 | 1 | 1 | **9** |
| **So et al., 2019**[3] | 1 | 0 | 1 | 1 | 1 | 1 | 1 | 1 | 0 | **7** |
| **Mohamed et al., 2022** [4] | 1 | 0 | 0 | 1 | 1 | 1 | 1 | 1 | 0 | **6** |
| **Al Sairafi & Mohamed, 2009** [5] | 1 | 1 | 0 | 1 | 1 | 1 | 1 | 1 | 1 | **8** |
| **Saadoon et al., 2014** [6] | 1 | 1 | 0 | 1 | 1 | 1 | 1 | 1 | 1 | **8** |
| **Al Nsour et al., 2012** [7] | 1 | 1 | 1 | 1 | 1 | 1 | 1 | 1 | 1 | **9** |
| **Al Rifai & Nakamura, 2015** [8] | 1 | 1 | 1 | 1 | 1 | 1 | 1 | 1 | 1 | **9** |
| **Bou-Orm et al., 2018** [9] | 1 | 1 | 1 | 1 | 1 | 1 | 1 | 1 | 1 | **9** |
| **Obeidat et al., 2012** [10] | 1 | 1 | 1 | 1 | 1 | 1 | 1 | 1 | 1 | **9** |
| **Al-Meer et al., 2011** [11] | 1 | 1 | 1 | 1 | 1 | 1 | 1 | 1 | 1 | **9** |
| **Aldohaian et al., 2019** [12] | 1 | 1 | 1 | 1 | 1 | 1 | 1 | 1 | 1 | **9** |
| **Husamaldien & Ali S. Dauod., 2016**[13] | 1 | 0 | 1 | 1 | 1 | 1 | 1 | 1 | 1 | **8** |
| **Alsalmi & Othman, 2022**[14] | 1 | 1 | 1 | 1 | 1 | 1 | 1 | 1 | 1 | **9** |
| **Alwan et al., 2017**  [15] | 0 | 0 | 0 | 1 | 1 | 1 | 1 | 1 | 1 | **6** |
| **AMARIN et al., 2008** [16] | 1 | 1 | 1 | 1 | 1 | 1 | 1 | 0 | 1 | **8** |
| **Alshamlan et al., 2023** [17] | 1 | 0 | 1 | 1 | 1 | 1 | 1 | 1 | 1 | **8** |
| **Hasan et al., 2021** [18] | 1 | 0 | 1 | 1 | 1 | 1 | 1 | 1 | 1 | **8** |
| **Alzahrani et al., 2018** [19] | 1 | 0 | 1 | 1 | 1 | 1 | 1 | 1 | 1 | **8** |
| **Fadhil et al., 2008** [20] | 1 | 1 | 0 | 1 | 1 | 1 | 1 | 0 | 1 | **7** |
| **Elgamal, 2015** [21] | 1 | 0 | 0 | 1 | 1 | 1 | 1 | 1 | 1 | **7** |
| **Zahid et al., 2022** [22] | 1 | 1 | 1 | 1 | 1 | 1 | 1 | 1 | 1 | **9** |
| **Abdulmalek & Kalary, 2019** [23] | 1 | 0 | 1 | 1 | 1 | 1 | 1 | 1 | 1 | **8** |
| **Altunkurek et al., 2022**[24] | 1 | 1 | 1 | 1 | 1 | 1 | 1 | 1 | 1 | **9** |
| **Dhaher, 2019** [25] | 1 | 0 | 1 | 1 | 1 | 1 | 1 | 1 | 1 | **8** |
| **Alshahrani MS, 2020**[26] | 1 | 1 | 1 | 1 | 1 | 1 | 1 | 1 | 1 | **9** |
| Mwanje J, 2023 (27) |  |  |  |  |  |  |  |  |  |  |
| **Barghouti FF, 2008** [28] | 1 | 0 | 1 | 1 | 1 | 1 | 1 | 1 | 1 | **8** |
| **Ibrahim et al., 2022** [29] | 1 | 0 | 1 | 1 | 1 | 1 | 1 | 1 | 1 | **8** |
| **Al-Attar et al., 2014** [30] | 0 | 0 | 0 | 0 | 1 | 1 | 1 | 1 | 1 | **5** |
| **Heena et al., 2019** [31] | 1 | 0 | 0 | 1 | 1 | 1 | 1 | 1 | 1 | **8** |
| **Yahyai et al., 2021** [32] | 1 | 1 | 1 | 1 | 1 | 1 | 1 | 1 | 1 | **9** |
| **Bencherit et al., 2022** [33] | 1 | 0 | 1 | 1 | 1 | 1 | 1 | 1 | 1 | **8** |
| **Al Kalbani et al., 2022** [34] | 1 | 0 | 0 | 1 | 1 | 1 | 1 | 1 | 1 | **7** |
| **El-Hammasi et al., 2009** [35] | 1 | 1 | 0 | 1 | 1 | 1 | 1 | 1 | 1 | **8** |
| **Al Eyd & Shaik, 2012** [36] | 1 | 1 | 0 | 1 | 1 | 1 | 1 | 1 | 0 | **7** |
| **Almobarak et al., 2016** [37] | 1 | 0 | 1 | 1 | 1 | 1 | 1 | 1 | 1 | **8** |
| **Alnafisah et al., 2019** [38] | 1 | 0 | 1 | 1 | 1 | 1 | 1 | 0 | 1 | **7** |
| **Rezq et al., 2023**[39] | 1 | 0 | 1 | 1 | 1 | 1 | 1 | 1 | 1 | **8** |
| **Walz et al., 2022** [40] | 1 | 0 | 1 | 1 | 1 | 1 | 1 | 1 | 1 | **8** |
| **Arechkik et al., 2023** [41] | 1 | 1 | 1 | 1 | 1 | 1 | 1 | 1 | 1 | **9** |
| **Ghamdi, 2022** [42] | 1 | 0 | 1 | 1 | 1 | 1 | 1 | 1 | 1 | **8** |
| **Akkour et al., 2021**[43] | 1 | 0 | 1 | 1 | 1 | 1 | 1 | 1 | 0 | **7** |
| **Bendahhou et al., 2023** [44] | 1 | 1 | 1 | 1 | 1 | 1 | 1 | 1 | 1 | **9** |
| **Gamaoun, 2018** [45] | 1 | 1 | 1 | 1 | 1 | 1 | 1 | 1 | 1 | **9** |
| **Alshammiri, 2022** [46] | 1 | 0 | 1 | 1 | 1 | 1 | 1 | 1 | 1 | **8** |
| **Telvizian et al., 2021** [47] | 1 | 0 | 1 | 1 | 1 | 1 | 1 | 1 | 1 | **8** |
| **Alshehri H et al., 2024** [48] | 1 | 1 | 1 | 1 | 1 | 1 | 1 | 1 | 1 | **9** |
| **Alfareh M et al., 2024** [49] | 1 | 1 | 1 | 1 | 1 | 1 | 1 | 1 | 1 | **9** |
| **M Al Kindi R et al., 2024** [50] | 1 | 0 | 1 | 1 | 1 | 1 | 1 | 1 | 1 | **8** |
| **Atef S et al., 2024** [51] | 1 | 0 | 1 | 1 | 1 | 1 | 1 | 1 | 1 | **8** |
| **Alkhamis et al., 2023** [52] | 1 | 1 | 1 | 1 | 1 | 1 | 1 | 1 | 1 | **9** |
| **Elbarazi et al., 2023** [53] | 1 | 1 | 1 | 1 | 1 | 1 | 1 | 1 | 1 | **9** |
| **Prashanth et al., 2023**[54] | 1 | 1 | 1 | 1 | 1 | 1 | 1 | 1 | 1 | **9** |
| **Al-Shamsi et al., 2023**[55] | 1 | 1 | 1 | 1 | 1 | 1 | 1 | 1 | 1 | **9** |

**Fig. S1: Meta-analysis of the** **overall rate of cervical cancer screening uptake in Arab countries**

**Fig. S2: Sensitivity analysis using the leave-one-out method for the overall cervical cancer screening uptake rate in Arab countries.**

**References**

1. Al-Amro SQ, Gharaibeh MK, Oweis AI. Factors Associated with Cervical Cancer Screening Uptake: Implications for the Health of Women in Jordan. Infect Dis Obstet Gynecol. 2020;2020.

2. Jassim G, Obeid A, Al Nasheet HA. Knowledge, attitudes, and practices regarding cervical cancer and screening among women visiting primary health care Centres in Bahrain. BMC Public Health. 2018;18.

3. So VHT, Channon AA, Ali MM, Merdad L, Al Sabahi S, Al Suwaidi H, et al. Uptake of breast and cervical cancer screening in four Gulf Cooperation Council countries. European Journal of Cancer Prevention. 2019;28:451–6.

4. Mohamed ML, Tawfik AM, Mohammed GF, Elotla SF. Knowledge, Attitude, and Practice of Cervical Cancer Screening, and HPV Vaccination: A Cross-Sectional Study Among Obstetricians and Gynecologists in Egypt. Matern Child Health J. 2022;26:565–74.

5. Al Sairafi M, Mohamed FA. Knowledge, attitudes, and practice related to cervical cancer screening among Kuwaiti women. Med Princ Pract. 2009;18:35–42.

6. Saadoon OZ, Amin RM, Jadoo SAA. Factors Influencing Pap Smear Practice Among Primary School Teachers In Diyala City, Iraq. Malaysian Journal of Public Health Medicine. 2014;:19–28.

7. Al Nsour M, Brown DW, Tarawneh M, Haddadin R, Walk H. Breast and cervical cancer screening among women in Jordan: Findings from the behavioural risk factor surveillance system - 2007. Open Breast Cancer Journal. 2012;4:1–7.

8. Al Rifai R, Nakamura K. Differences in Breast and Cervical Cancer Screening Rates in Jordan among Women from Different Socioeconomic Strata: Analysis of the 2012 Population-Based Household Survey. Asian Pac J Cancer Prev. 2015;16:6697–704.

9. Bou-Orm IR, Sakr RE, Adib SM. Cervical cancer screening among Lebanese women. Rev Epidemiol Sante Publique. 2018;66:1–6.

10. Obeidat BR, Amarin ZO, Alzaghal L. Awareness, practice and attitude to cervical Papanicolaou smear among female health care workers in Jordan. Eur J Cancer Care (Engl). 2012;21:372–6.

11. Al-Meer FM, Aseel MT, Al-Khalaf J, Al-Kuwari MG, Ismail MFS. Knowledge, attitude and practices regarding cervical cancer and screening among women visiting primary health care in Qatar. East Mediterr Health J. 2011;17:855–61.

12. Aldohaian AI, Alshammari SA, Arafah DM. Using the health belief model to assess beliefs and behaviors regarding cervical cancer screening among Saudi women: a cross-sectional observational study. BMC Womens Health. 2019;19.

13. Husamaldien LG, Ali S. Dauod. knowledge, awareness and practices about breast and cervical cancer in a group of women in Erbil city–Iraq. Tikrit medical Journal. 2016;21:54–66.

14. Alsalmi SF, Othman SS. Cervical Cancer Screening Uptake and Predictors Among Women in Jeddah, Saudi Arabia. Cureus. 2022;14.

15. Alwan NA, Al-Attar WM, Al Mallah N, Abdulla KN. Assessing the Knowledge, Attitude and Practices Towards Cervical Cancer Screening Among a Sample of Iraqi Female Population | Iraqi journal of biotechnology. 2017. https://jige.uobaghdad.edu.iq/index.php/IJB/article/view/87. Accessed 5 Dec 2023.

16. AMARIN ZO, BADRIA LF, OBEIDAT BR. Attitudes and beliefs about cervical smear testing in ever-married Jordanian women. EMHJ-Eastern Mediterranean Health Journal. 2008;14:389–97.

17. Alshamlan NA, Alomar RS, Alabdulkader AM, Alghamdi FA, Aldakheel AA, Al Shehri SA, et al. Beliefs and Utilization of Cervical Cancer Screening by Female Health Care Workers in Saudi Arabia Using the Health Belief Model: A Nationwide Study. Int J Womens Health. 2023;15:1245–59.

18. Hasan T, Jwad Taher T, Ghazi H. AWARENESS REGARDING PAP SMEAR AMONG WOMEN IN BAGHDAD CITY, IRAQ. Wiad Lek. 2021;LXXIV:2287–92.

19. Alzahrani HH, Thabet HA, Zahrani A Al. Knowledge, Attitudes and Practices in Relation to Cervical Cancer Screening among Female Employees at King Abdulaziz University. International journal of Nursing Didactics. 2018;8:01–10.

20. Fadhil I, Baqer B, Hlwachi F, Ahmed E, Karim N, Reefy S. Factors associated with cervical cancer knowledge and practice among Bahraini women. Asian Pac J Trop Med. 2008;1:72–8.

21. Elgamal S. Knowledge, Attitude and Practices of Nurses Regarding Cervical Cancer and Human Papilloma Virus Vaccine in Tanta City. 2015.

22. Zahid HM, Qarah AB, Alharbi AM, Alomar AE, Almubarak SA. Awareness and Practices Related to Cervical Cancer among Females in Saudi Arabia. Int J Environ Res Public Health. 2022;19.

23. Abdulmalek IY, Kalary KM. Knowledge, Attitude and Practice about Pap Smear among Married Women Attending Primary Health Care Centers in Duhok City. International Journal of Medical Research & Health Sciences. 2019;8:139–53.

24. Altunkurek ŞZ, Mohamed SH, Şahin E, Yilmaz S. Knowledge and attitudes of healthcare professionals working in a training and research hospital on early diagnosis of cervical cancer (a Somalia example): cross-sectional study. BMC Womens Health. 2022;22:1–10.

25. Dhaher EA. Knowledge, Attitudes and Practices of Women in the Southern Region of Saudi Arabia Regarding Cervical Cancer and the Pap Smear Test. Asian Pac J Cancer Prev. 2019;20:1177.

26. Alshahrani MS SSAA. Awareness and Attitude to the Risk of Cervical Cancer and Screening Method among Women in the Najran Region of Southern Saudi Arabia. International Journal of Medicine in Developing Countries. 2020;4:2299–304.

27. Cervical Cancer Screening in resource-poor settings of South Sudan: Access Coverage, Associated Factors and Health Syst…. https://ouci.dntb.gov.ua/en/works/4MYJw1j4/. Accessed 3 Jan 2024.

28. Barghouti FF TAFE. Awareness and behavior about Pap smear testing in family medicine practice. Saudi Med J . 2008;29:1036–40.

29. Ibrahim HA, Nahari MH, Al-Thubaity DD, Alshahrani MA, Elgzar WT, El Sayed HA, et al. Saudi women health beliefs and associated factors regarding cervical cancer prevention at Najran city: A theory-based study. Afr J Reprod Health. 2022;26:43–51.

30. Al-Attar W, Alwandawi N, Algomele H. Knowledge, Attitude and Practice of Workers about The Cervical Cancer and Pap Smear in The College of Nursing. Al-Kufa Journal for Nursing Sciences. 2014;4.

31. Heena H, Durrani S, Alfayyad I, Riaz M, Tabasim R, Parvez G, et al. Knowledge, Attitudes, and Practices towards Cervical Cancer and Screening amongst Female Healthcare Professionals: A Cross-Sectional Study. J Oncol. 2019;2019.

32. Yahyai T Al, Raisi M Al, Kindi R Al. Knowledge, Attitudes, and Practices Regarding Cervical Cancer Screening among Omani Women Attending Primary Healthcare Centers in Oman: A Cross-Sectional Survey. Asian Pac J Cancer Prev. 2021;22:775.

33. Bencherit D, Kidar R, Otmani S, Sallam M, Samara K, Barqawi HJ, et al. Knowledge and Awareness of Algerian Students about Cervical Cancer, HPV and HPV Vaccines: A Cross-Sectional Study. Vaccines (Basel). 2022;10.

34. Al Kalbani R, Al Kindi R, Al Basami T, Al Awaisi H. Cervical Cancer-related Knowledge and Practice among Omani Women Attending a Family Medicine and Public Health Clinic. Oman Med J. 2022;37:e374.

35. El-Hammasi K, Samir O, Kettaneh S, Al-Fadli A, Thalib L. Use of and attitudes and knowledge about pap smears among women in Kuwait. J Womens Health (Larchmt). 2009;18:1825–32.

36. Al Eyd GJ, Shaik RB. Rate of Opportunistic Pap Smear Screening and Patterns of Epithelial Cell Abnormalities in Pap Smears in Ajman, United Arab Emirates. Sultan Qaboos Univ Med J. 2012;12:473.

37. Almobarak AO, Elbadawi AA, Elmadhoun WM, Elhoweris MH, Ahmed MH. Knowledge, Attitudes and Practices of Sudanese Women Regarding the Pap Smear Test and Cervical Cancer. Asian Pac J Cancer Prev. 2016;17:625–30.

38. Alnafisah RA, Alsuhaibani RA, Alharbi MA, Alsohaibani AA, Ismai AA. Saudi Women’s Knowledge and Attitude toward Cervical Cancer Screening, Treatment, and Prevention: A Cross-Sectional Study in Qassim Region (2018-2019). Asian Pac J Cancer Prev. 2019;20:2965.

39. Rezq KA, Algamdi M, Alanazi R, Alanazi S, Alhujairy F, Albalawi R, et al. Knowledge, Perception, and Acceptance of HPV Vaccination and Screening for Cervical Cancer among Saudi Females: A Cross-Sectional Study. Vaccines (Basel). 2023;11.

40. Walz L, Mohamed D, Haibah A, Harle N, Al-Ali S, Moussa AA, et al. Knowledge, attitudes and practices concerning breast cancer, cervical cancer and screening among healthcare professionals and students in Mogadishu, Somalia: a cross-sectional study. Ecancermedicalscience. 2022;16.

41. Arechkik A, lahlou L, Kharbach A, Baba MA, Obtel M, Razine R. Awareness, Knowledge and Attitude Regarding Cervical Cancer among Women Living with HIV in the Souss-Massa Region, Southern Morocco: A Cross-Sectional Study. Asian Pac J Cancer Prev. 2023;24:2875.

42. Ghamdi NH Al. Knowledge of human papilloma virus (HPV), HPV-vaccine and pap smear among adult Saudi women. J Family Med Prim Care. 2022;11:2989.

43. Akkour K, Alghuson L, Benabdelkamel H, Alhalal H, Alayed N, AlQarni A, et al. Cervical Cancer and Human Papillomavirus Awareness among Women in Saudi Arabia. Medicina (B Aires). 2021;57.

44. Bendahhou K, Serhier Z, Diouny S, Ouadii K, Barkouk A, Niyonsaba A, et al. Women’s Knowledge and Attitudes Towards Cervical Cancer Screening in Morocco. Cureus. 2023;15.

45. Gamaoun R. Awareness and knowledge about cervical cancer prevention methods among Tunisian women. J Prev Med Hyg. 2018;59:E30.

46. Alshammiri SM. Knowledge and attitudes of cervical cancer screening among female high school teachers in Hail city: A cross-sectional study. J Family Med Prim Care. 2022;11:6390.

47. Telvizian T, Al Ghadban Y, Alawa J, Mukherji D, Zgheib NK, Sawaf B, et al. Knowledge, beliefs, and practices related to cancer screening and prevention in Lebanon: Community and social media users’ perspectives. European Journal of Cancer Prevention. 2021;30:341–9.

48. Alshehri HD, Alqudah O, Almadani FB, Aldalbahi AM, Jarrah O a, Albashaireh A. Factors Associated With the Uptake of Cervical Cancer Screening Among Family Medicine Physicians, Compared With Women of the Community in Riyadh, Saudi Arabia. Cureus. 2024;16.

49. Alfareh M, Obeid D, Alhoshan H, Basri R, Alhamlan FS. Low uptake of Pap test as a cervical cancer screen among Saudi women: Findings from a national survey. International Journal of Gynecology & Obstetrics. 2024;00:1–8.

50. M Al Kindi R, H Al Sumri H, M Al Muhdhoori T, Al Mamari K, A Al Kalbani M, H Al-Azri M. Knowledge of cervical cancer screening among Omani women attending a university teaching hospital: a cross-sectional study. BMC Womens Health. 2024;24:1–9.

51. Atef S, Taha E, Reda ;, Elmouafy I, Mohamed MA, Mai ;, et al. Awareness, Beliefs and Behaviors of Women About Cervical Cancer Screening at Primary Health Care Centers in Port Said City. Port Said Scientific Journal of Nursing. 2024;11:268–92.

52. Alkhamis FH, Alabbas ZAS, Mulhim JE Al, Alabdulmohsin FF, Alshaqaqiq MH, Alali EA. Prevalence and Predictive Factors of Cervical Cancer Screening in Saudi Arabia: A Nationwide Study. Cureus. 2023;15:e49331.

53. Elbarazi I, Alam Z, Abdullahi AS, Al Alawi S, AlKhanbashi M, Rabaa A, et al. Knowledge, Attitudes and Practices of Women in the UAE Towards Breast and Cervical Cancer Prevention: A Cross-Sectional Study. Cancer Control. 2023;30.

54. Cervical Cancer Risk Factor Awareness and Utilization of Screening Program among Women in United Arab Emirates. https://journalononcology.org/articles/joo-v3-1100.html. Accessed 31 Oct 2024.

55. Humaid Al-Shamsi S, Humaid Al-Shamsi A, Humaid Al-Shamsi M, Sajwani A, Alzaabi MS, Al Hammadi O, et al. The Perception and Awareness of the Public about Cancer and Cancer Screening in the United Arab Emirates, a Population-Based Survey. Clinics and Practice 2023, Vol 13, Pages 701-714. 2023;13:701–14.
